# Management of Giant Frontoethmoid Osteoma by Combined Approach

**DOI:** 10.7759/cureus.47046

**Published:** 2023-10-15

**Authors:** Neizekhotuo B Shunyu, Hanifa Akhtar, Anuradha Deka, Prachurya Tamuli, Prakash Deb

**Affiliations:** 1 Otorhinolaryngology, All India Institute of Medical Sciences, Guwahati, Guwahati, IND; 2 Otorhinolaryngology, North Eastern Indira Gandhi Regional Institute of Health and Medical Sciences, Shillong, IND; 3 Anesthesiology and Critical Care, All India Institute of Medical Sciences, Guwahati, Guwahati, IND

**Keywords:** ethmoidal sinus, frontal sinus, surgery, giant frontoethmoid osteoma, osteoma

## Abstract

Giant osteomas of the frontoethmoidal region often manifest early with ocular symptoms and intracranial complications. The management involves careful surgical planning of both the approach and reconstruction. In the present case report, a case of giant frontoethmoid osteoma presented with ocular symptoms and cosmetic deformity. The case was managed by a combined endoscopic and open surgical approach along with reconstruction of the sinus wall defect using a pericranial flap and titanium mesh. The outcome was found to be satisfactory with the resolution of ocular symptoms and good cosmesis.

## Introduction

Osteomas are common benign tumours primarily found in the craniofacial region, especially in the nasal and paranasal sinuses. The frontal and ethmoid sinuses are the most commonly affected sites [1-3]. The majority of osteomas remain asymptomatic and are managed conservatively. However, surgical intervention becomes necessary when osteomas cause symptoms [3,4]. An osteoma is considered a "giant" sinus osteoma when its diameter exceeds 30 mm [5]. The choice of surgical approach depends on the grading of the osteoma, its attachment, the availability of surgical facilities, and the expertise of the surgeon [1,2,5]. After the removal of frontal sinus osteomas, reconstruction of the anterior wall becomes necessary and can be achieved using various biomaterials and implants [6,7].

This report describes the management of a case of giant frontoethmoid osteoma with displacement of the globe through a combined approach by bicoronal incision and restoration of the contour of frontal sinus by titanium mesh and use of pericranial flap to cover the mesh, to prevent extrusion and improve postoperative forehead cosmesis.

## Case presentation

**Figure 1:**
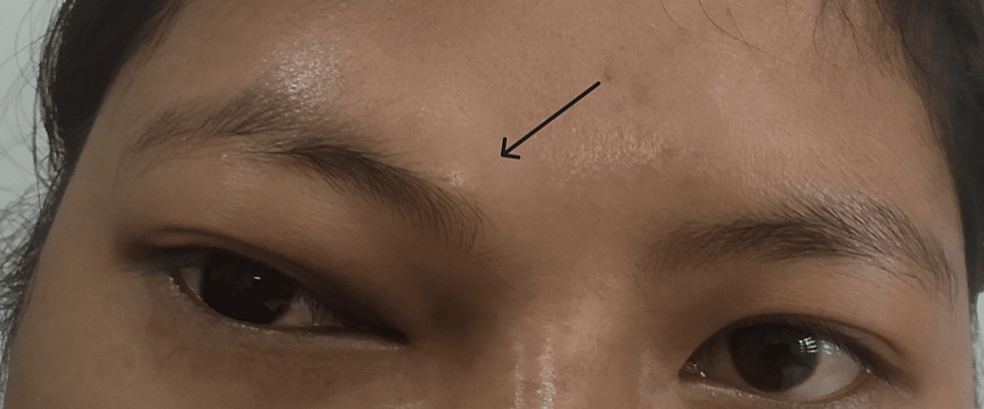
Preoperative picture of the patient showing swelling over the right medial part of the eyebrow and lateral displacement of the right eye (in straight gaze).

An early adolescent female presented at our outpatient department with pain over her right forehead for three months and swelling above her right eye for two months. The pain was sudden in onset, of throbbing type, gradually worsening, and originated from the medial end of the right eyebrow and radiated to the right forehead and face. Subsequently, she developed double vision. There was no history of nasal blockage or diminished vision. Clinical examination revealed a diffuse swelling around the medial part of the eyebrow (Figure 1). The swelling was firm to hard, non-tender, non-pulsatile, and irreducible with no local signs of inflammation. Eye examination showed normal visual acuity and extraocular movement. Nasal endoscopic examination was normal.

**Figure 2:**
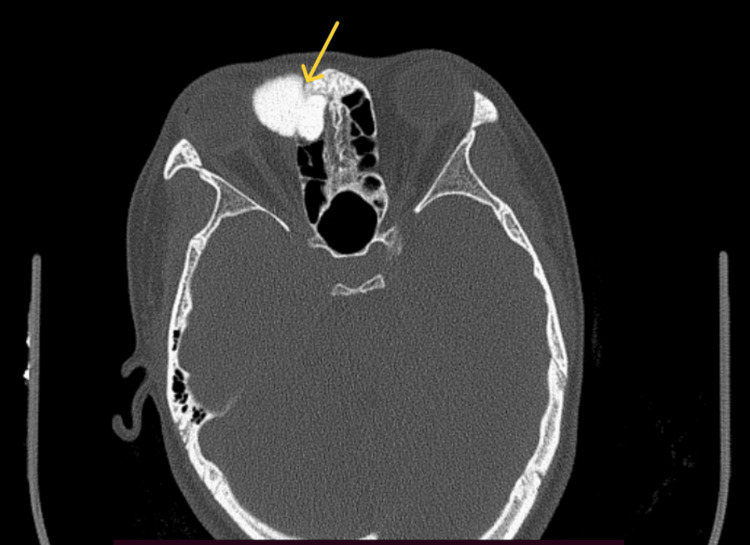
Axial view of CT PNS showing frontoethmoid osteoma causing mass effect with the displacement of the right globe. CT, Computed tomography; PNS, Paranasal sinus.

**Figure 3:**
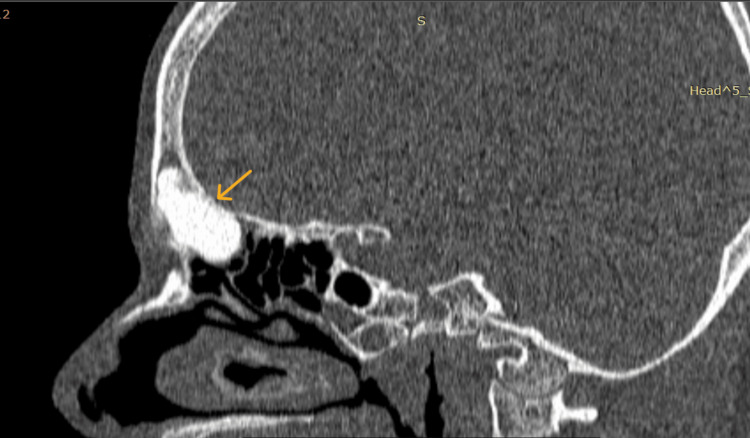
Sagittal section of CT PNS showing a well-defined, hyperdense bony density mass in the frontoethmoid region with broad-based attachment to the frontal bone and ethmoid roof with adjacent frontal bone thinning and endosteal scalloping. There was no evidence of the mass extending into the intracranial region, and the posterior wall of the frontal sinus appeared intact. CT, Computed tomography; PNS, Paranasal sinus.

**Figure 4:**
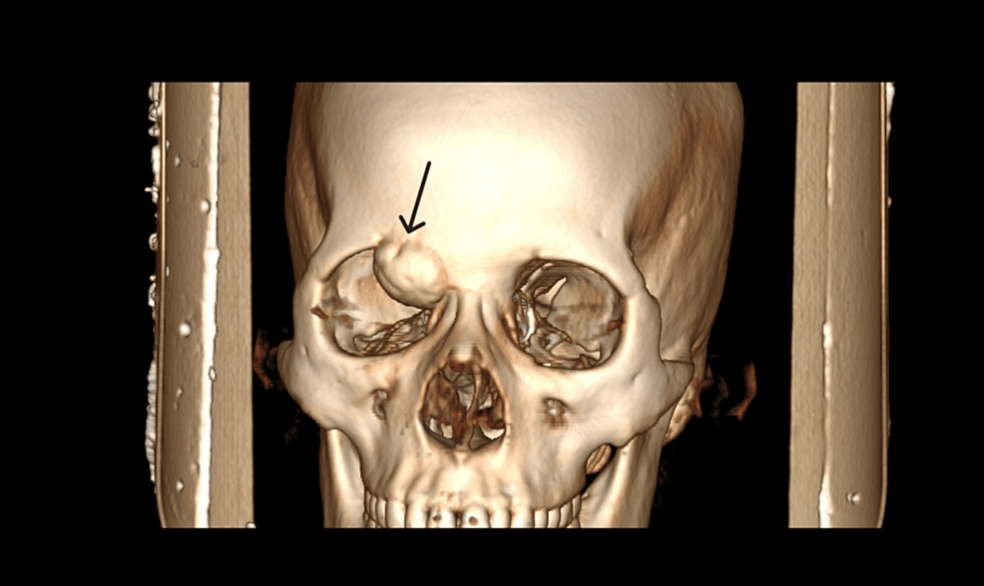
Surface volume rendering of the maxillofacial area showing osteoma involving the roof of the right orbit and floor of the right frontal sinus.

A computed tomography (CT) scan of the paranasal sinuses and skull base revealed a well-defined, hyperdense bony density mass in the frontoethmoid region with broad-based attachment to the frontal bone and ethmoid roof, causing displacement of the right globe. Its measurements were 3.2 cm x 2.6 cm x 2.6 cm, with adjacent frontal bone thinning and endosteal scalloping. There was no evidence of the mass extending into the intracranial region, and the posterior wall of the frontal sinus appeared intact (Figures 2, 3, 4).

**Figure 5:**
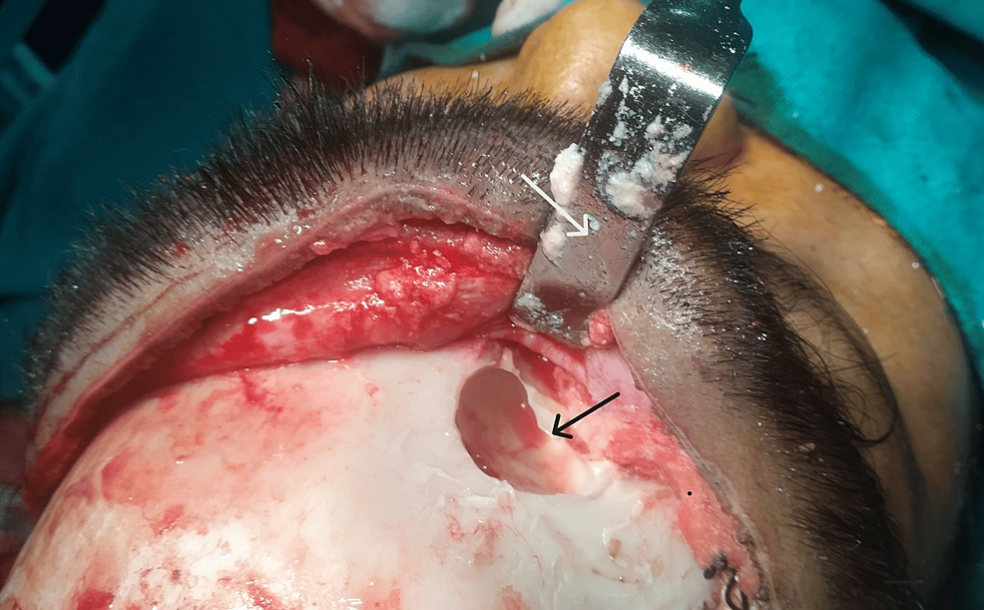
Intraoperative photo showing the defect of the anterior wall of the frontal sinus and inside of the right frontal sinus (black arrow) after removal of osteoma. The retractor (white arrow) retracting the galeopericranial flap anteriorly to provide adequate exposure of the frontal sinus.

After obtaining the patient's consent, surgery by combined open and endoscopic approach was planned as the tumour had attachment at the frontal and ethmoidal sinuses. A bicoronal incision was made posterior to the hairline in subgaleal plane anteriorly till supraorbital rim. Anteriorly based scalp flap was raised and the tumour was resected completely using a drill and curette. The posterior wall of the frontal sinus was found to be intact, but a minimal protrusion of orbital fat was observed within the frontal sinus cavity (Figure 5). The endoscopic approach involved anterior and posterior ethmoidectomy to establish the outflow tract. The anterior frontal wall defect was closed using a titanium mesh followed by the placement of a pericranial flap over the titanium mesh. Histopathological examination confirmed the diagnosis of osteoma.

**Figure 6:**
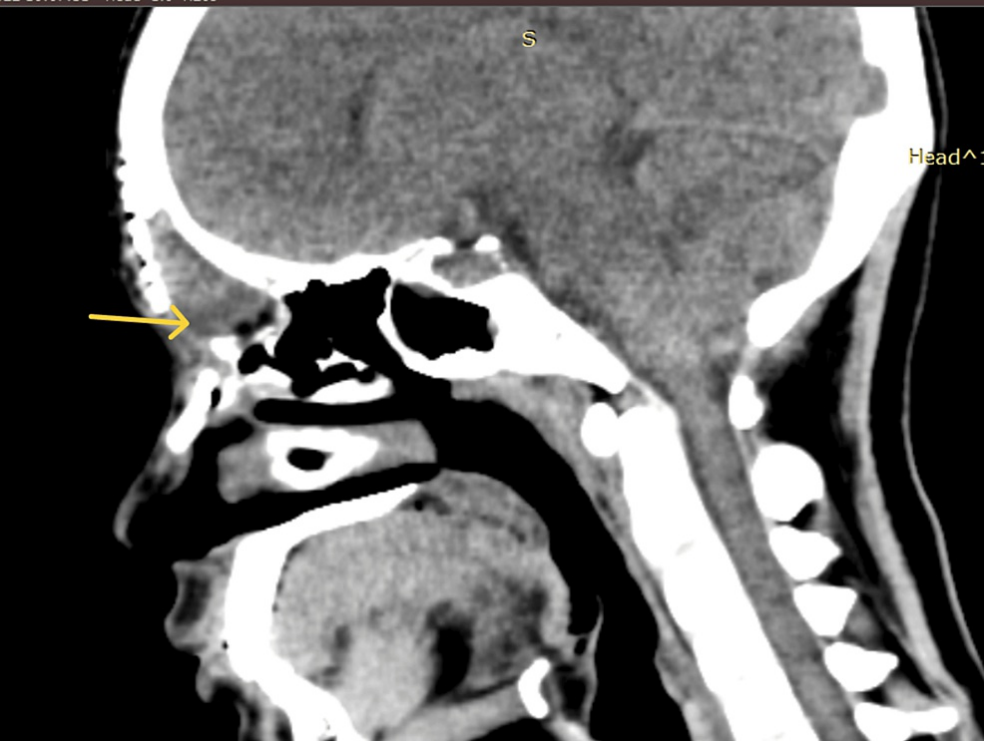
Sagittal view of CT PNS done on the 11th postoperative day showing a defect in the anterior bony wall of the right frontal sinus with collection in the sinus cavity. CT, Computed tomography; PNS, Paranasal sinus.

**Figure 7:**
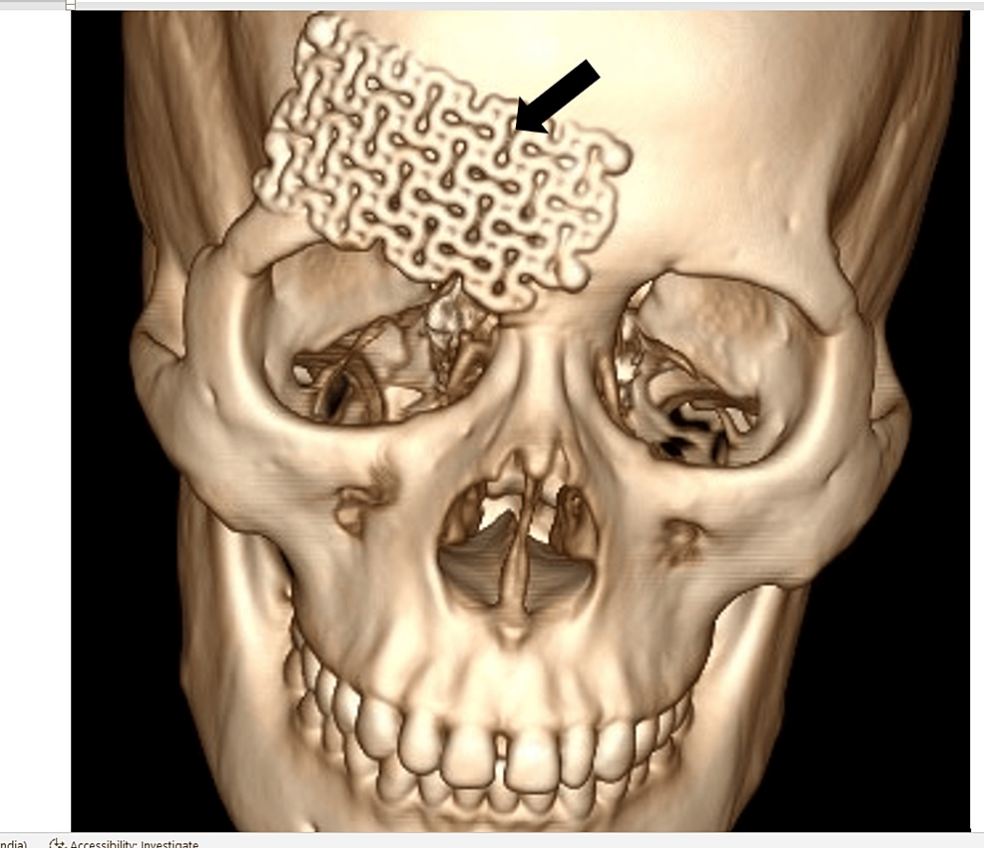
3D reconstruction volume rendering (VR) image showing titanium mesh (black arrow) over the anterior wall of the right frontal sinus, used to cover the defect and improve cosmesis.

**Figure 8:**
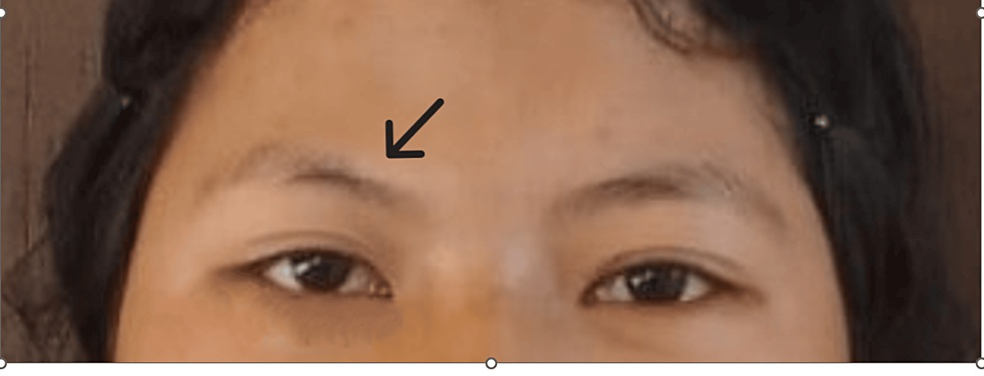
Frontal photograph of the patient after one year of surgery. There is an improvement of gaze and diplopia. There is no visible scar over the face (the operated site is shown with a black arrow).

The postoperative period was uneventful. A CT scan done on the 11th day showed a frontal sinus defect with the presence of mesh, but no residual tumour was observed (Figures 6, 7). The surgical scar healed well, resulting in a satisfactory cosmetic appearance and improvement in double vision (Figure 8).

## Discussion

Most osteomas found in the paranasal sinuses grow slowly and remain asymptomatic [1,2,4]. However, a subset of osteomas may present with clinical symptoms depending on their size and location. The most common symptom is a progressive frontal headache caused by sinus drainage obstruction, facial pain and deformity [1]. Additionally, osteomas can lead to symptoms related to ocular and intracranial involvement [1,4,5]. Ocular symptoms may be excessive tearing, eye bulging, double vision, vision loss, and orbital cellulitis [5,6]. Osteomas in the frontoethmoidal region can cause early ocular symptoms due to their close proximity to the orbit [5,6]. Giant osteomas are associated with both ocular and intracranial complications [4-6]. In our case of giant frontoethmoid osteoma, symptoms progressed in a very short time due to the involvement of both the frontal and ethmoidal sinuses. Moreover, due to the osteoma's grade 4 (Chiu’s staging), there was throbbing, severe frontal pain due to obstruction of sinus drainage along with swelling and double vision. Thus, patients' clinical course matches with tumour characteristics and literature.

Surgical intervention is recommended for osteomas that exhibit symptoms, progress rapidly and obstruct frontal recess [1,4-8]. Different classification exists for staging frontal osteomas and serves as a guide for selecting the most suitable approach, but there is a lack of uniformity in guidelines [9,10]. The surgical approach may be open surgery, endoscopic surgery, or a combination of both. Chiu staging classifies frontal osteomas as grade I-IV and recommends endoscopic resection of small frontal osteomas medial to the sagittal line passing through the lamina papyracea (grade I and II) while using the external approach for grade III and IV osteomas. Grade III osteomas are those whose base of attachment is anteriorly or superiorly located within the frontal sinus and/or whose tumour extends lateral to a virtual sagittal plane through the lamina papyracea and grade IV osteomas are when the tumour fills the entire frontal sinus [9]. Watley et al. also introduced a classification system for frontal osteomas, which takes into account factors such as attachment, favourable or unfavourable anatomy, and extra sinus involvement [10]. In cases such as ours, where there is a giant osteoma, multicompartment osteoma with broad-based attachment, or a high-grade osteoma, the combined approach continues to be the preferred choice [11,12].

An external approach can be made through either an eyebrow incision or a bicoronal incision [1,6]. Eyebrow incision poses risks to the supraorbital nerve and results in a visible facial scar. In contrast, the bicoronal incision is preferred for larger tumours, those with intracranial extension or orbital involvement as it offers good exposure, a barely noticeable scar, and also gives an opportunity to harvest galeal or pericranial flaps [1,6,12,13].

A variety of methods and materials can be used to repair the contour defect resulting from anterior frontal bone table resection like autologous skull bone, titanium mesh, porous polyethylene, methyl methacrylate, and plastic polymers [6,7,13]. The flexibility and strength of titanium mesh offer distinct benefits. It not only offers precise contouring, provides robust support to restore the frontal sinus defect, effectively prevents displacement but also results in excellent cosmetic outcomes [7,13].

Additionally, patients may experience skin texture changes, such as scars or dimples on the forehead, as a late complication due to the formation of adhesions between the skin-soft tissue layer and underlying hardware and these can be avoided by using pericranial flap in combined or external approach for management of frontal osteomas [14]. The use of pericranial flaps has been widely employed for the management of anterior skull base tumours and fractures [15,16], and in the management of frontal osteoma [13]. Its utilization can provide multiple benefits, including sinus obliteration and the safeguarding of hardware used in the reconstruction of the anterior wall of the frontal sinus, thus reducing the risk of postoperative complications and the need for additional surgery [13,14].

In our case, we chose a bicoronal incision to improve exposure and achieve favourable cosmetic outcomes. Moreover, as the incision site and implant site were different, the use of a bicoronal incision and pericranial flap prevented the extrusion of implants like titanium mesh in our case. The use of titanium mesh to close the anterior wall defect of the frontal sinus resulted in excellent cosmetic outcomes for our patient. All the patients should be followed up at 1, 3, 6 and 12 months and yearly thereafter, but in our case, the patient could not come for follow-up at regular intervals due to personal reasons.

Our study highlights the safe, effective method of the use of pericranial flap and titanium mesh for surgery of giant frontoethmoid osteoma but monitoring of both functional and aesthetic outcomes at follow-up is essential to understand the efficacy of these procedures in giant frontal or frontoethmoid osteomas.

## Conclusions

Giant osteomas of frontal and ethmoidal sinuses necessitate thorough planning of surgical approach. A bicoronal incision in giant frontoethmoid osteoma provides not only good exposure of the surgical field but also provides the advantage of harvesting pericranial flap. Pericranial flaps are versatile flaps that can be utilised in the management of frontal and ethmoid sinus osteomas by open or combined approach. Its utilization can provide multiple benefits, including sinus obliteration, safeguarding of titanium mesh and reducing the risk of postoperative complications and the need for additional surgery.
